# Epilepsy

**Published:** 1862-07

**Authors:** Edward Sutton Smith

**Affiliations:** 10 Union Square, New York


### EPILEPSY.

AFTER THE FRENCH OF HERPIN.

BY EDWARD SUTTON SMITH, M.D.

The idea generally entertained by the profession, that epilepsy is incurable; the prevalence of the disease in the United States, and its *curability* in France, have led me to translate the salient points of M. Herpin's celebrated work, with some additions drawn from my own experience in the treatment of this malady in America.

During a long residence in the French capital, and from a personal acquaintance with M. Herpin, who has now devoted more than thirty years to this disease *alone*, and whose practice is not only very great, but successful, I satisfied myself that epilepsy could be treated in the same manner, and with the same happy results, in this country. With the above slight introduction, I offer the following article, one of a series of two or three that I propose to write for the *American Medical Monthly*.

To show the estimation in which the work is held by the profession in France, I append the report of the committee chosen by the Institute to examine into its merits.

INSTITUTE OF FRANCE, *December*, 16*th*, 1850.

*Report.*—"Dr. Herpin, of Geneva, has forwarded, under the title of Practical Studies in the Prognosis and Treatment of Epilepsy, a work composed, first, of thirty-eight observations relative to epilepsy, collected by the author; then an estimate of the different circumstances of these thirty-eight observations. These original materials serve as a basis, enabling the author to give, upon many of the symptoms of epilepsy, new views, and to determine the value of these symptoms from the double point of view of the prognosis and diagnosis of epilepsy. He also makes use of these same facts to study and compare the influence which every condition of age, sex, constitution, as well as different antecedent or concomitant diseases, may exercise upon the greater or less gravity of epilepsy, and the possibility of its cure. He again studies, under the same conditions, the good or bad influence which may be exercised by hereditary descent, menstruation, pregnancy, the married or single state, the degree of individual intelligence, social position, and finally, the length of time that the disease has existed. The importance of the subject studied by Dr. Herpin, the severity of the method which he has followed, in order to observe and arrange the facts, and lastly, the interest of many of the results which he has attained, have appeared to the Commission to merit a reward of fifteen hundred francs."

Every reflecting physician, when present at the cure of any disease, is compelled to demand what has been the influence of the treatment he advised. The reply, with the exception of a small number of cases free from difficulties, is environed with some doubt, unless the physician be endowed with a philosophic spirit. Consequently, this doubt, up to a certain point, surrounds all the precepts that direct practical medicine. By what means can we cause this uncertainty to disappear, and elevate therapeutics to a true science? We have already remarked, whatever may be the number of successful results, that some special means has procured you in the treatment of a disease, you cannot affirm the utility of your course, and still less, place any value upon it, except in proportion as you can show that this disease, if left to itself, would have had different and less favorable results. In theory, the means of comparison can only be furnished by a natural and complete series of facts where the affection was left entirely to the efforts of nature. Now, there are but few cases where this purely passive observation is possible. Thus, forced to have resource to more direct means in the midst of agents indefinitely varied and often contradictory among themselves, that are employed in diseases, it is not presumable that if some are effacious, others are inert, and some hurtful? Thus, may we not conclude that a numerous series of facts, selected without reference to the methods of treatment, will give results which, admitting them to be a little more favorable than those of simple expectation, will not differ in any very marked manner? Consequently, may we not hope that at some future day we shall have a standard in each disease which shall serve as a common measure for all therapeutics upon the same disease? We have never doubted that this hope could be realized at some future day, for science already holds the elements of this work for a certain number of diseases. Meanwhile, nothing approaches nearer the wished-for guage than the comparative examination of opinions that have already been given upon the prognosis of a disease by a number of practical authors, chosen in different places and at different times, among the most skilful and conscientious.

The complete absence of statistical information upon the prognosis of epilepsy obliges us to resort to the latter means; fortunately, this affection is one of those where gravity, when left to itself, is so well known, that exactitude will not be required.

The prognosis is either general or special.

It is *general* when limited to the calculation of ordinary chances, the cure, and mortality of a disease. The general prognosis of an affection is obtained by combining the results of a series of numerous facts. These results, particularly if they are expressed in numerals, will serve to show the actual merit of any treatment that has been employed in an entirely different series.

The questions that may be raised in the general prognosis of epilepsy, it appears to us, may be reduced to the three following:

Can epilepsy be spontaneously cured; and if so, what is the ordinary proportion of these cures?

Has medical skill any influence upon the favorable termination of this malady, and what are the limits of this influence?

In incurable cases, what are the results of this affection?

1st. We shall find in the history of medicine incontestible proofs that epilepsy has cured itself, though this fortunate result appears somewhat rare, according to Maisonneuve—about 4 in 100.

2d. According to the opinion of authors, it is well known that medical treatment has cured a certain number of cases of epilepsy.

3d. Without furnishing us with any very exact results in the cure of incurable epilepsy, our historical documents warrant our asserting that this malady is not incompatible with the prolongation of life, but that it shortens its length, altering more or less profoundly the intellectual and moral faculties.

Of the three questions that we have above mentioned, that which is the most important for us is the first, which treats of the spontaneous cure of epilepsy. We have seen that this result is quite rare, and estimating it at the rate of 4 in 100, we shall not be far from the truth.

In our examination, we shall consider separately two partial series of our own; the 38 cases of our first series we divide, according to their termination, as follows:—

*First Category.*—Three cases where the treatment was only followed two or three days. One of these patients left my clinic; the two others remained epileptic, one being an idiot.

*Second Category—First Section.*—Two patients who were not treated, but spontaneously cured; one having had attacks for five years; the other, subject to turns of vertigo, after having but one in four years, has not had another for four years.

*Second Section.*—Seventeen cases that were treated, which is exactly half of the 33 patients that were more or less under treatment.

*Third Category—First Section.*—Three patients whose condition was ameliorated, though they only followed an incomplete treatment, and would probably have been cured had they been more persevering, judging from the results produced by the remedy when taken in an insufficient manner.

*Second Section.*—Four individuals in whom the treatment having been properly followed, evidently extended the interval between the attacks without causing them to disappear.

*Fourth Category.*—Nine cases, all obstinate.

The figures in these four categories afford us the following conclusions:—

1st. Of 38 epileptics, 2 were cured without the aid of art; which gives the proportion of spontaneous cures in 19 cases, of about 5 in 100, which differs but little from the facts stated by Maisonneuve—4 in 100.

2d. Of 33 epileptics that were treated, 17, or 51 in 100, were cured; 7, or 21 in 100, were ameliorated; 3 of these by an incomplete treatment; 9, or 27 in 100, experienced no benefit under the influence of prolonged and varied treatment.

Thus, the number of our cures (without taking into consideration those two who were ameliorated, two of whom, at least, very nearly approximated the cured,) is one half. But the more these results differ from the opinion of the immense majority of practitioners, the more necessary it will be to answer in advance those objections that will be raised against these conclusions.

No one will dispute our obstinate cases; let us leave for a moment those who were ameliorated, and examine those cases that were cured. To these it may be objected:—

That our diagnosis is wanting in exactness.

That our patients were cured by the interposition of nature alone.

That our cures were only greater or less suspensions of the accession, bearing in mind the returns which we call relapses.

1st. As to the exactness of our diagnosis, we merely ask any impartial man to look at the notes that accompany our observations, and read the chapter on the classification of the paroxysms. They will see that we have carefully studied the distinction between epilepsy, cerebral congestion, hysteria, etc.

2d. To those who merely see spontaneous cures in our cases, we place before them the prognosis of epilepsy, as given by all authors, without exception.

3d. This only remains, and will be the most specious objection of those who regard our cures as a simple postponement of the attacks. We will place before them a synopsis of our cases. Let us commence by the cases to the number of eleven, where there was no relapse.

*Observation* 7.—Duration four years; constant vertigoes every day; four attacks in three months, three of which took place in eight days; many suspensions by treatment during four years, the longest of which was eight months. Absolute cure, which has lasted *more than thirteen years*—(30th Nov. 1837, to the end of Sept. 1850.)

*Observation* 8.—Duration, five months; at the close many are daily. Cure, which has lasted *more than twelve years*—(March 13th, 1838, to the end of September, 1850.)

*Observation* 11.—Attacks during three months, many times monthly before the treatment. Cure, which has lasted *more than six years*—(August 16th, 1844, to the end of September, 1850.)

*Observation* 14.—Many paroxysms daily for fifteen days. Cure, which has lasted *more than five years*—(June 24th, 1845, to the end of September, 1850.)

*Observation* 15.—Two paroxysms and several vertigoes for a month. Cure, which has lasted *about five years*—(October 25, 1845, to the end of September, 1850.)

*Observation* 10.—Vertigoes for ten years, many in a month. Cure, which has lasted *about five years*—(November, 1845, to the end of September, 1850.)

*Observation* 20.—One single attack. Cure, which has lasted *five years*—(September 30th, 1846, to the end of September, 1850.)

*Observation* 22.—One single attack. Cure, which lasted *till the death of the patient, more than three years*—(October 14th, 1846, to November 13th, 1849.)

*Observation* 6.—Attacks for ten years; greatest interval, two months. Cure, which lasted *till the death of the patient, twenty-seven months*—(December 27th, 1840, till the 16th of March, 1843.)

*Observation* 18.—About one attack a week for three months; greatest interval, fifteen days. Cure, which lasted *till the death of the patient, more than five months*—(October 7th, 1849, to March 2d, 1850.)

If any one will compare, in the case of those patients that have not died, the time that has elapsed since the last accession up to the close of my observations, with the ordinary interval of their attacks, it does not seem to me possible that any person can suppose that these are mere postponements in the course of the disease.

Let us now see if our cases of relapse are not really cures. They are six in number.

*Observation* 9.—Frequent vertigoes. Cure, lasting *seven years.* Relapse: more and more frequent vertigoes for a year, then an attack. Cure, (excepting a few turns of vertigo, which have ceased for the past four years,) which has lasted *more than nine years*—(September 30th, 1841, to September, 1850.)

*Observation* 12.—Three attacks in three months. Cure, which has lasted *six years.* Relapse: two consecutive attacks. Cure, which lasted *five years and a-half*—(March 16th, 1845, to the end of September, 1850.)

*Observation* 13.—Three incomplete attacks in sixteen months. Cure, which lasted *twenty-six months.* Relapse: a single accession. Cure, which has lasted *six years and a-half*—(February 19th, 1844, till September, 1850.)

*Observation* 48.—Commenced at the age of 16 years, (1812,) five years before the first menstruation. For six years, one or two attacks of epilepsy a week. Once had forty-eight in twenty-four hours, the consequence of bad treatment. At long intervals, some attacks of hysteria. Suspension of the attacks for three years, owing to some treatment of whose composition she is ignorant. Relapse, (1812): for six years, two of three attacks every week; the longest interval being three weeks, (not counting three pregnancies, during which they completely ceased.) Attacks were more frequent at the menstrual periods. From 1828 to 1829, not a single accession: then two paroxysms of attacks in eight months. For for years and a-half, isolated attacks, usually returning at an interval of about a year or eighteen months. In July, 1835, complete relapse, in consequence of a year's imprisonment: for ten months, attacks almost daily; some ten or twelve in the same day. Six months after this relapse, I treated her four months. Cure, which, with the exception of two consecutive attacks in September, 1836, lasted till the death of the patient from an ovarian cyst, the 2d of August 1850, *fourteen years* after the last attack.

*Observation* 62.—When this young girl came to my clinic, she had already more than 1200 attacks, preceded, for five years, by frequent vertigoes, that had been gradually converted into acces; these latter had, in their turn, been transformed into attacks, and are not comprised in the above account. During the first five months she was under my treatment, the number of attacks was exactly two a day. To-day (December 24, 1851), this young girl, after having had only eight attacks in seven months, has been completely free from them for more than four months. Though half idiotic at the time of her first visit, she now enjoys the full use of all her faculties.

Out of 48 patients,

| | | |
|---|---|---|
| 26 were cured | 54 in | 100 |
| 10 ameliorated | 21 | " |
| 12 incurable | 25 | " |

From all which we deduce the result:—

1st. That generally epilepsy is not spontaneously cured, nor does it take place in a twentieth part of the cases.

2d. That medical skill can benefit three-quarters of those who suffer from this terrible malady; that it can *cure* more than one-half, and produce a greater or less amelioration in a fifth part of the cases; and that the proportion of incurable cases is only one-quarter.

*The criterion for epilepsy if found in the total number of attacks or accessions that the patient has had.*

*In the case of patients that have not had vertigo, or if they have not been very frequent, nor lasted more than ten years, we may be almost certain of effecting a cure.*

*As regards the attacks and accessions, the prognosis is entirely favorable, if the number has been less than* 100.

From 100 to 500 the chances are less favorable.

*The prognosis is unfavorable if the attacks have been more than* 500, *and in such cases a cure can only be regarded as an exceptional case.*

If it can be established that recent epilepsy is almost always curable, to render humanity an immense service, it is only necessary that the world should know the power of medical skill when taken in time; and every physician should be convinced, that with confidence, exactitude, and perseverance, in the great majority of cases he may be assured of success. To attain this double object, every influential man, whatever his sphere of action, from this time forth, should endeavor, as far as epilepsy is concerned, to popularize the advice given by the poet:—

Principiis obsta, sero medicina paratur,
Cum mala per longas invaluere moros.

10 Union Square, New York. —*Amer. Med. Monthly.*

